# Morbidity and mortality among very preterm singletons following fertility treatment in Australia and New Zealand, a population cohort study

**DOI:** 10.1186/s12884-017-1235-6

**Published:** 2017-02-02

**Authors:** Alex Y Wang, Abrar A. Chughtai, Kei Lui, Elizabeth A. Sullivan

**Affiliations:** 1 Faculty of Health, University of Technology Sydney, PO Box 123, Broadway, NSW 2007 Australia; 2 School of Public Health and Community Medicine, University of New South Wales, Sydney, NSW 2031 Australia; 3 School of Women’s and Children’s Health, University of New South Wales, Sydney, NSW 2031 Australia

**Keywords:** Preterm birth, Very preterm birth, Assisted reproductive technology, Hyper-ovulation, Artificial insemination

## Abstract

**Background:**

Due to high rates of multiple birth and preterm birth following fertility treatment, the rates of mortality and morbidity among births following fertility treatment were higher than those conceived spontaneously. However, it is unclear whether the rates of adverse neonatal outcomes remain higher for very preterm (<32 weeks gestational age) singletons born after fertility treatment. This study aims to compare adverse neonatal outcomes among very preterm singletons born after fertility treatment including assisted reproductive technology (ART) hyper-ovulution (HO) and artificial insemination (AI) to those following spontaneous conception.

**Methods:**

The population cohort study included 24069 liveborn very preterm singletons who were admitted to Neonatal Intensive Care Unit (NICU) in Australia and New Zealand from 2000 to 2010. The in-hospital neonatal mortality and morbidity among 21753 liveborn very preterm singletons were compared by maternal mode of conceptions: spontaneous conception, HO, ART and AI. Univariate and multivariate binary logistic regression analysis was used to examine the association between mode of conception and various outcome factors. Odds ratio (OR) and adjusted odds ratio (AOR) and 95% confidence interval (CI) were calculated.

**Results:**

The rate of small for gestational age was significantly higher in HO group (AOR 1.52, 95% CI 1.02–2.67) and AI group (AOR 2.98, 95% CI 1.53–5.81) than spontaneous group. The rate of birth defect was significantly higher in ART group (AOR 1.71, 95% CI 1.36–2.16) and AI group (AOR 3.01, 95% CI 1.47–6.19) compared to spontaneous group. Singletons following ART had 43% increased odds of necrotizing enterocolitis (AOR 1.43, 95% CI 1.04–1.97) and 71% increased odds of major surgery (AOR 1.71, 95% CI 1.37–2.13) compared to singletons conceived spontaneously. Other birth and NICU outcomes were not different among the comparison groups.

**Conclusions:**

Compared to the spontaneous conception group, risk of congenital abnormality significantly increases after ART and AI; the risk of morbidities increases after ART, HO and AI. Preconception planning should include comprehensive information about the benefits and risks of fertility treatment on the neonatal outcomes.

## Background

The latest report of Australia’s mothers and babies shows that 25113 of the 301810 babies (8.3%) born in Australia in 2012 were preterm (<37 weeks gestational age), the most common cause of death among infants [1]. Worldwide, around 14.9 million babies were born preterm in 2010 (11.1% of total birth in the same year). Of these, about 5% were extreme preterm (<28 weeks), 11% were very preterm (28–31 weeks gestational age) and 84% moderate to late preterm (32–36 weeks gestational age) [2]. In comparison, of preterm births in Australia in 2012, 11% were extreme preterm, 9% were very preterm and 80% were moderate to late preterm [1]. Evidence shows that extreme preterm and very preterm births are at increased risk of severe morbidity and mortality compared to moderate preterm births (32–36 weeks) and term births (>36 weeks) [3].

With the advanced care in neonatal intensive care units (NICU), the survival of very preterm babies has been improved in recent years, especially in developed countries. The 2012 annual report by the Australian and New Zealand Neonatal Network (ANZNN) shows that the survival rate before NICU discharge was 70% for births of 24 weeks gestational age and 98% for births of 31 weeks gestational age [4]. The ANZNN data also shows that the NICU survival rates varied by plurality, with significantly higher survival rates for singletons than for multiples.

The literature suggests that multiple birth is the most significant risk factor of preterm birth and subsequent adverse neonatal outcome [5]. Preterm birth occurred in 60.8% of twins and in 94.8% of higher order multiple births compared to 6.9% of singletons [1]. The neonatal death rates of twins (10.9 per 1,000 live births) and higher order multiples (28.7 per 1,000 live births) were significantly higher than that of singletons (2.1 per 1,000 live births) [1]. Given the higher rate of multiple pregnancy following assisted reproductive technology (ART), births following ART were at increased risk of preterm birth and subsequent adverse neonatal outcomes. Previous studies also reported increased morbidity and mortality among births following ART compared to those following spontaneous conception [6, 7]. However, it is unclear whether increased risk of subsequent adverse neonatal outcomes among very preterm singletons is related to ART treatment itself or more attributable to the underlying subfertility [8]. The study using a population cohort approach aims to compare adverse neonatal outcomes among very preterm singletons born after fertility treatment including ART, hyper-ovulation (HO) and artificial insemination (AI) to those born following spontaneous conceptions. We hypothesized that very preterm singletons following fertility treatment have increased risk of morbidity and mortality.

## Methods

### Data

This study used data and definitions from the ANZNN data collection. The ANZNN is collected annually from all NICUs in Australia and New Zealand. Liveborn babies included in ANZNN are either born at less than 32 weeks gestation, or weighed less than 1,500 g at birth, or those who received assisted ventilation or major surgery (surgery that involved opening a body cavity). A research dataset including all liveborn singletons (*N* = 24,069) of <32 weeks gestation born between 2000 and 2010 was supplied from ANZNN for this study. Of these, 17696 (73.5%) were with birthweight < 1500 g, 9854 (40.9%) required assisted ventilation and 1946 (8.1%) had major surgery.

### Main outcome measures

The primary outcomes are morbidity and mortality before NICU discharge. Outcome measures were categorised into conditions at birth and NICU complications. Conditions at birth include small for gestational age (SGA, <10^th^ percentile for the gestation), 5 min APGAR score (less than 7 was categorised as moderate/severe depressed), extreme low birth weight (extreme LBW; <1000 g), intubation during resuscitation and presence of congenital abnormalities (defined as structural abnormalities including deformations that are present at birth and diagnosed prior to separation from care). SGA for non-ART singletons were estimated from already published birthweight for gestational age percentile charts [9, 10]. SGA for ART singletons was estimated using published birthweight percentiles by gestational age for ART births [11]. NICU complications include hyaline membrane disease, necrotizing enterocolitis (NEC), intraventricular haemorrhage, retinopathy of prematurity, major surgery and death.

### Comparison group

Conditions at birth and NICU complications were compared among liveborn singletons by four modes of conceptions flagged the ANZNN database: spontaneous conception (no fertility treatment used for this pregnancy), HO (any hormone therapy used to stimulate ovulation), ART (any method of in-vitro handling oocyte or embryos including in-vitro fertilisation, gamete intra- fallopian transfer, zygote intra fallopian transfer) and AI. ANZNN data collection does not have detailed information about the ART fertilisation procedures, fresh or frozen embryos, and the number of embryo transferred and stage of embryo development (blastocyst or cleavage stage). Other study factors include maternal age, ethnicity, gestational age and maternal complications, including pregnancy inducted hypertension in pregnancy (A systolic blood pressure > 140 mmHg and/or diastolic blood pressure > 90 mmHg, or a rise in systolic blood pressure > 25 mmHg and/or a rise in diastolic blood pressure > 15 mmHg from a reading before conception or in 1^st^ trimester; confirmed by 2 readings 6 h apart), antepartum haemorrhage (Significant haemorrhage in the time from 20 weeks gestation to the end of second stage of labour) and premature rupture of membranes (Confirmed spontaneous rupture of membranes occurring prior to the onset of labour and before 37 weeks gestation).

### Statistical analysis

Demographics characteristics (maternal age, aboriginal status, gestational age, previous preterm birth and previous perinatal death) and other maternal conditions (premature rupture of membranes, pregnancy inducted hypertension, antepartum haemorrhage and antenatal steroid) were compared among mode of conception and differences in the means and proportions were tested. Analysis of variance was used for continuous variables and Chi-square test was used for categorical variables. Univariate and multivariate binary logistic regression analysis was used to examine the association between mode of conception and various outcome factors. Odds ratio (OR) and adjusted odds ratio (AOR) (adjusted for maternal age, gestational age, ethnicity, previous pre term, previous prenatal death, maternal hypertension, antepartum haemorrhage, PROM and antenatal steroid) and 95% CI were calculated. The level of significance was set at 0.05, and 95% CIs were used to minimize the risk of chance findings. Statistical Package for Social Sciences (SPSS, Inc., Chicago, IL, USA Version 21) was used for data analysis.

## Results

**Fig. 1:**
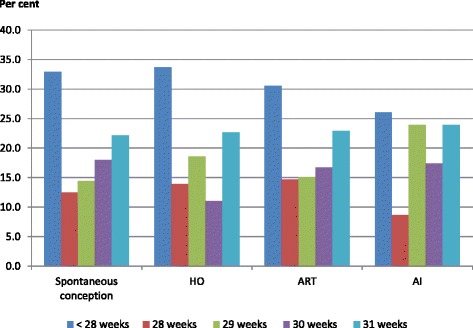
Distribution of gestational age of very preterm and extreme preterm singletons by mode of conceptions

The information on mode of conception was available for 21,753 (90.4%) singletons. Among these, 94.4% (20,530/21,753) of singletons were born following spontaneous conception, 4.4% (953/21,753) after ART, 1% (216/21,753) after HO, 0.2% (54/21,753) after AI. Figure 1 presents the distribution of gestational age by the mode of conceptions. The proportion of extreme preterm was 26.1% in AI group, compared to 30.6% in ART group, 33.7% in HO group and 32.9% in spontaneous conception group.

The average age of mother following ART (34.6 ± 4.7 years), AI (33.3 ± 4.7 years) and HO (30.2 ± 4.9 years) was significantly higher compared to those who conceived spontaneously (28.9 ± 6.3 years) (*p* < 0.01). Similarly, the mean gestational age was significantly different among four groups (*p* = 0.01) (Table 1). Compared to the spontaneously conceived singletons, mothers of ART singletons had higher proportions of pregnancy inducted hypertension and use of antenatal steroids (*p* < 0.01).

**Table 1:** Demographics of very preterm singletons by mode of conceptions

	Spontaneous		HO		ART		AI		*P* value^a^
	(20,530)		(216)		(953)		(54)		
	#	%	#	%	#	%	#	%	
Maternal age (years) Mean ± SD	28.87	±6.3	30.36	±4.9	34.64	±4.7	33.33	±4.7	<0.05^b^
Gestational age (weeks) Mean ± SD	28.44	±2.5	28.12	2.4±	28.25	2.3±	28.19	2.3±	0.01
Aboriginal status									
Yes	1274	6.2	4	1.9	10	1.0	0	0.0	<0.05
no	17079	83.2	186	86.1	830	87.1	52	96.3	
Not stated	2177	10.6	26	12	113	11.9	2	3.7	
Previous preterm birth									
Yes	3928	19.1	24	11.1	78	8.2	7	13.0	<0.05
No	16506	80.4	192	88.9	874	91.7	47	87.0	
Not stated	96	0.5	0	0.0	1	0.1	0	0.0	
Previous perinatal death									
Yes	1239	6.0	11	5.1	38	4.0	5	9.3	<0.05
No	19218	93.6	205	94.9	915	96.0	49	90.7	
Not stated	73	0.4	0	0.0	0	0.0	0	0.0	
Premature rupture of membranes									
Yes	5243	25.5	44	20.4	246	25.8	10	18.5	0.301
No	15256	74.3	171	79.2	704	73.9	44	81.5	
Not stated	31	0.2	1	0.5	3	0.3	0	0.0	
Pregnancy inducted hypertension									
Yes	3619	17.6	58	26.9	199	20.9	20	37.0	<0.05
No	16895	82.3	157	72.7	752	78.9	34	63.0	
Not stated	16	0.1	1	0.5	2	0.2	0	0.0	
Antepartum haemorrhage									
Yes	5028	24.5	47	21.8	262	27.5	13	24.1	0.063
No	15488	75.4	168	77.8	689	72.3	41	75.9	
Not stated	14	0.1	1	0.5	2	0.2	0	0.0	
Antenatal steroid									
None	2724	13.3	17	7.9	76	8	3.0	5.6	<0.05
Incomplete	5514	26.9	56	25.9	234	24.6	13	24.1	
Complete	9377	45.7	111	51.4	468	49.1	33	61.1	
More than 7 days	2548	12.4	31	14.4	156	16.4	5	9.3	
Not stated	367	1.8	1	0.5	19	2.0	0	0.0	
Gender of baby									
Male	11279	54.9	122	56.5	517	54.2	33	61.1	0.98
Female	9233	45.0	94	43.5	436	45.8	21	38.9	
Ambiguous	2	0.0	0	0.0	0	0.0	0	0.0	
Not stated	16	0.1	0	0.0	0	0.0	0	0.0	

^a^ chi square test.

^b^ Analysis of variance

**Fig. 2:**
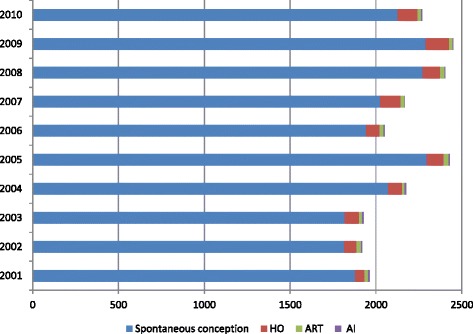
Number of very preterm and extreme preterm singletons by mode of conceptions 2001–2010

Figure 2 shows the number of singletons admitted to NICU by the mode of conceptions over the period of 10 years. NICU admissions for the singletons conceived spontaneously and after ART treatment increased by 11.7% and 48.8% respectively from 2001–10.

The rates of adverse birth outcomes were higher in HO, ART and AI singletons, compared to the spontaneous conception. In the univariate analysis, HO was associated with SGA, and extreme LBW; ART was associated with extreme LBW, intubation during resuscitation and major malformation; and AI was associated with SGA and congenital abnormalities (Table 2). Compared to very preterm singletons conceived spontaneously, the odds of SGA was about 3 times higher for those after AI (AOR 2.98, 95% CI 1.53–5.81) and 1.5 times higher for those after HO (AOR 1.52, 95% CI 1.02–2.67). Similarly, the odds of major abnormalities was 3 times (AOR 3.01, 95% CI 1.47–6.19) higher for AI singletons and 1.7 times (AOR 1.71, 95% CI 1.36–2.16) higher for ART singletons than spontaneous singletons. Other birth outcomes were not significantly different between spontaneous conception group and the three fertility treatment groups.

**Table 2:** Birth conditions of very preterm singletons by mode of conceptions

	Number	Percent	OR (95% CI)	AOR (95% CI)^a^
Small for gestational age				
Spontaneous conception	1833	8.9	Ref	Ref
HO	35	16.2	**1.97 (1.37–2.84)**	**1.52 (1.02–2.67)**
ART	102	10.7	1.22 (0.99–1.51)	1.09 (0.87–1.38)
AI	16	29.6	**4.29 (2.39–7.71)**	**2.98 (1.53–5.81)**
5 min APGAR (Mod/ severe depressed)				
Spontaneous conception	3659	18.0	Ref	Ref
HO	35	16.2	0.88 (0.61–1.27)	0.78 (0.52–1.18)
ART	176	18.6	1.04 (0.88–1.23)	1.02 (0.85–1.24)
AI	10	18.5	1.04 (0.52–2.06)	1.07 (0.52–2.22)
Extreme Low birth weight (<1000 g)				
Spontaneous conception	6676	32.5	Ref	Ref
HO	88	40.7	**1.43 (1.09–1.87)**	1.13 (0.72–1.79)
ART	351	36.8	**1.21 (1.06–1.38)**	1.11 (0.88–1.40)
AI	22	40.7	1.43 (0.83–2.46)	1.01 (0.43–2.42)
Intubation during resuscitation				
Spontaneous conception	8405	41.0	Ref	Ref
HO	101	46.8	1.26 (0.97–1.65)	1.16 (0.84–1.60)
ART	440	46.3	**1.24 (1.09–1.41)**	1.11 (0.94–1.30)
AI	22	40.7	0.97 (0.57–1.70)	0.87 (0.46–1.64)
Congenital abnormalities				
Spontaneous conception	1197	6.0	Ref	Ref
HO	10	4.9	0.80 (0.42–1.51)	0.80 (0.42–1.52)
ART	93	10.1	**1.75 (1.40–2.19)**	**1.71 (1.36–2.16)**
AI	9	16.7	**3.12 (1.52–6.40)**	**3.01 (1.47–6.19)**

^a^ Adjusted for maternal age, gestational age, ethnicity, previous pre term, previous prenatal death, maternal hypertension, antepartum haemorrhage, premature rupture of membranes and antenatal steroid

Significant results are in bold

The multivariate analysis shows that very preterm singletons born after ART have 43% higher odds of having NEC (AOR 1.43, 95% CI 1.04–1.97) and 71% higher odds of having major surgery (AOR 1.71, 95% CI 1.37–2.13) compared to those conceived spontaneously. Data stratification shows that major surgery was closely related to congenital abnormalities. Major surgery was reported in 33% and 11% of ART singletons with and without congenital abnormalities respectively (*p* value <0.05). Other NICU outcomes were not significantly different spontaneous conception group and the three fertility treatment groups (Table 3).

**Table 3:** NICU outcomes of very preterm singletons by mode of conceptions

	Number	Percent	OR (95% CI)	AOR (95% CI)^a^
Hyaline membrane disease				
Spontaneous conception	14574	71.9	Ref	Ref
HO	154	72.0	1.00 (0.74–1.36)	0.87 (0.62–1.21)
ART	724	76.9	**1.30 (1.11–1.52)**	1.18 (0.99–1.40)
AI	38	72.1	0.93 (0.52–1.67)	0.75 (0.41–1.42)
Necrotizing enterocolitis				
Spontaneous conception	817	4.0	Ref	Ref
HO	3	1.4	0.34 (0.11–1.04)	0.33 (0.10–1.05)
ART	49	5.2	1.30 (0.97–1.75)	**1.43 (1.04–1.97)**
AI	2	3.7	0.92 (0.22–3.80)	0.85 (0.20–3.60)
Intra ventricular haemorrhage				
Spontaneous conception	4327	23.1	Ref	Ref
HO	53	26.0	1.17 (0.85–1.60)	1.23 (0.86–1.75)
ART	229	25.6	1.14 (0.98–1.33)	1.20 (1.00–1.43)
AI	12	23.1	0.99 (0.52–1.90)	1.21 (0.61–2.40)
Retinopathy of prematurity				
Spontaneous conception	3896	26.6	Ref	Ref
HO	49	31.6	1.27 (0.91–1.79)	1.04 (0.70–1.52)
ART	187	27.2	1.03 (0.87–1.22)	1.00 (0.82–1.24)
AI and other	10	23.3	0.83 (0.41–1.70)	0.99 (0.44–2.17)
Major surgery				
Spontaneous conception	1642	8.1	Ref	Ref
HO	23	10.7	1.37 (0.89–2.11)	1.27 (0.79–2.02)
ART	122	12.9	**1.70 (1.39–2.06)**	**1.71 (1.37–2.13)**
AI	6	11.1	1.43 (0.61–2.34)	1.00 (0.389–2.60)
Deaths				
Spontaneous conception	1852	9.0	Ref	Ref
HO	20	9.3	1.02 (0.65–1.63)	0.67 (0.38–1.18)
ART	98	10.3	1.15 (0.93–1.43)	1.11 (0.86–1.42)
AI	9	16.7	2.01 (0.98–4.13)	2.10 (0.93–4.76)

^a^ Adjusted for maternal age, gestational age, ethnicity, previous pre term, previous prenatal death, maternal hypertension, antepartum haemorrhage, premature rupture of membranes and antenatal steroid

Significant results are in bold

## Discussion

This bi-national population study showed that the rates of some adverse neonatal outcomes are significantly increased among the very preterm singletons following fertility treatment. Compared to the spontaneous conception group, ART and AI groups had 1.7 times and 3.0 times increased odds of major malformation. Very preterm singletons following HO and AI had 1.5 times and 3.0 times increased odds of SGA than spontaneous conception group. ART is associated with 43% and 71% higher odds of NEC and major surgery compared to spontaneous conception group.

The literature shows that fertility treatments including ART, AI, HO and use of Clomiphene are associated with increased risk of adverse pregnancy and perinatal outcomes [12]. Very preterm and preterm birth are such adverse perinatal outcomes associated fertility treatment, and are leading causes of other morbidity and mortality [13, 14]. We selected very preterm singletons as our study population which reduced the confounding and interaction effects between the fertility treatment and adverse perinatal outcomes due to multiple pregnancies and prematurity. Agreed with the literature, our findings suggested that very preterm singletons following ART and other fertility treatment are at increased risk of fetal and neonatal outcomes [15–18].

A number of studies reported higher incidence of low birthweight among births following fertility treatment than spontaneous births [6, 19–22]. Since our study population was limited to very preterm singletons, the low birthweight is not a measure relevant to our study population. Instead, we used SGA to measure birthweight outcomes. Babies are SGA if their weights are below the 10th percentile for their gestational ages. Although the majority babies born SGA catch up in growth at 2 years old, SGA babies are at increased risk of morbidity and mortality [7]. Apart from genetic reasons, SGA is related to fetal, maternal and placental conditions. Subfertility, one of the maternal conditions is associated with increased risk of SGA [23]. Since all couples who access ART treatment have some level of subfertility, they are more likely to have a SGA baby [17]. Zhu and colleagues also reported a high rate of SGA in sub-fertile women regardless of ART treatment [23]. Even for very preterm singletons, those born to mothers following fertility treatment, an indicator of subfertility, have a higher rate of SGA than those following spontaneous conceptions.

Congenital anomaly is the one of the leading causes of neonatal death among the preterm babies [24]. Many studies have reported an increased rate of congenital anomalies among births following fertility treatment compared to spontaneous births [18, 25, 26], with the prevalence of congenital anomalies ranges from 4–9% among ART births according to various studies [12, 17, 27], and 4–6% in general population [28, 29]. Even though our rates are comparable with other published studies, it should be interpreted with cautions as we only included very preterm births in this study. Increase risk of congenital abnormality may be due to generally increased risk of congenital amorality in ART group or due to shift of gestational duration toward lower values among a normal rate of congenital amorality. Moreover, congenital anomalies may be on the casual pathway to low birth weight or preterm birth [30, 31].

Presence of congenital abnormalities is one of the major causes of surgery and other adverse neonatal outcomes in the neonatal period [32]. The most common reasons for major surgery in our study include vascular system (418 cases), skeletal system (151 cases), gastrointestinal (146 cases), genital tract (136 cases) and respiratory (105 cases). The higher rate of major surgery among ART singletons in our study is likely due to associated congenital abnormalities. Rates of major surgery were significantly in ART singletons with congenital abnormalities, compared to ART singletons without congenital abnormalities (*p* value <0.05). Similarly we need to assume that a chain of event might happen and in most cases birth conditions are related to the common NICU outcomes including NEC, intraventricular haemorrhage, hyaline membrane disease and retinopathy of prematurity among the very preterm babies [24]. Although the rates of all conditions were high in the ART group, only NEC was significantly associated with the ART in this study.

Necrotizing Enterocolitis is one of the most common severe diseases among preterm births, with high morbidity and mortality. Yee and colleagues suggested that the mortality for NEC can be up to 50% and 20–40% cases may need surgical treatment [33]. The rates of NEC in our study were 5.2% among ART and 4.0% among spontaneous conceived very preterm singletons. These are within the range between 3% and 15% reported by other studies [34]. The increase rate of NEC and very preterm ART singletons in our study remains unclean as the cause necrotizing enterocolitis is incompletely understood. Neu and Walker suggested that the cause of NEC is multifactorial, but it is preventable by withholding enteral feedings, using enteral antibiotics, feeding with expressed breast milk, and administering probiotic agents [35].

Death rates in ART treatment groups were high in our study, compared to the spontaneous conception group, however the difference was not statistically significant. In a previous NICU study in Australia, low mortality was observed in the ART twins and triplets compare to spontaneously conceived twins and triplets and authors attributed it “protective effect” to the “dichorionic pregnancies” and specialized care offered to the ART mothers [20]. Another study reported low rate of death among the babies born after the ART treatment and authors attributed it to the single embryo transfer practices and comparison of ART healthy babies with sick non ART babies [24]. However both these studies included multiple pregnancies in the analysis and there is ample of evidence suggests that ART singletons have high mortality compared to non-ART singletons [21].

Survival rates of the very preterm babies have been improved in the last few decades due to increase use of ventilator support, steroids and surfactants [36 ,37]. However the outcome is still poor among the babies born after the fertility treatment. It is not clear whether this is due to complication of treatment such as congenital abnormalities or due to maternal conditions such as pregnancy induced hypertension. These babies are at risk of long term neurological and behavioural complications which are not well studied [38]. The outcome in the very preterm singletons also may be different as it is associated with the gestational age. Among the preterm babies, the mortality is generally very high among the those born at 23 weeks (84%) compared to those born at 28 week (13%) [39]. The potential risk of very preterm birth and subsequent neonatal morbidity and mortality should be explained to the women undergoing ART treatment.

There are some limitations of this study, which are important while interpreting the results. In the ANZNN database four categories are mentioned under the flag “Assisted conception”: spontaneous conception, HO, ART and AI. It is possible that some HO and AI singletons were misreported into spontaneous conception group given the small number of HO and AI singletons were identified in this study. However, we are unable to track the assisted conception from other records. Similarly we are unable to track the pregnancies characterises including gestational sacs and fetal hearts. It is suggested that singletons born as a result of vanish twins/triples have increased risk of adverse perinatal outcomes those born with initial one gestational sac/fetal heart (Wang et al. 2009). This is especially relavent to HO and ART singletons where multiple gestational sacs pregnancies are prevalent [12, 14].

ART included complex procedures such as oocyte collection, ICSI procedure, cleavage stage transfer, frozen embryo transfer, and number of embryos transferred. Detailed information on types of cycle, ART procedures, and embryo transfers which may be associated with adverse maternal and fetal outcomes is not available in the database [40, 41]. For example high rate of congenital abnormalities are reported after the ICSI procedure [12] and cleavage stage transfer [42]. Perinatal outcomes are usually favourable following frozen embryo transfers than fresh embryo transfers [13, 43]. Similarly, double embryo transfer is related to multiple birth [44] and multiple births had higher chances of congenital abnormalities than the singletons [45]. Compared to the single embryo transfers, 1.5 fold increase in fetal death has been reported for births following double embryo transfers [14].

Another limitation of this study was restricting analysis to only extreme preterm and very (<32 weeks) births, which limits the study generalisation to all preterm births. ANZNN data collection includes births of gestational age < 32 weeks in Australia and New Zealand, however the evidence suggests that the rate of preterm in the developed countries are mainly increased for the moderate preterm birth (32–36 weeks gestation) [46]. As ANZNN only includes babies of birth weight <1,500 g, many LBW babies may not be admitted to NICU and were not included from the study. Comparison between babies of birth weight <1,500 g and those of birth weight 1500–2499 g is important since the later have less complications compared to very preterm babies. Another limitation of this study is that we are unable to identify women who have a history of subfertility but conceived spontaneously. These women were included in the spontaneous conception group although they were inherently different from women without history of infertility [47, 48]. Previous studies also show that maternal and childhood complications are more common in subfertile women compared to fertile women [49–51]. This indicates that some biological factors may play a role however there is limited evidence.

Our multivariate analysis was adjusted for maternal age, gestational age, ethnicity, previous pre term, previous prenatal death, maternal hypertension, antepartum haemorrhage, PROM and antenatal steroid. The residual confounding may exist as we were unable to adjust factors such as maternal smoking, BMI, location and size of NICU, method of delivery and parental social-economic status. Moreover, increase risk of congenital abnormality and other morbidities after fertility treatments in NICU setting may not be generalised to the all babies born after fertility treatment. We only examined morbidity and mortality among cases admitted to NICUs. Fetal deaths and terminations due to prenatally diagnosed congenital abnormality are not included. Finally the comparison was made between spontaneous conceived singletons and those following ART, HO and AI. We did not make comparison across fertility treatment groups due to small sample size in HO and AI groups. Further studies need be conducted by directly comparing outcomes in sub-fertile/infertile women conceived with or without fertility treatment and type of fertility treatment [52, 53].

## Conclusions

Very preterm (<32 weeks gestational age) singletons following HO, ART and AI had higher rates of some neonatal morbidity than spontaneous singletons. Compared to the spontaneous conception group, risk of birth defects significantly increases after ART and AI; the risk of morbidities increases after ART, HO and AI. Preconception planning should include comprehensive information about the benefits and risks of fertility treatment on the neonatal outcomes.

## Abbreviations

AI: Artificial insemination
ANZNN: Australian and New Zealand Neonatal Network
AOR: Adjusted odds ratio
ART: Assisted reproductive technology
CI: Confidence interval
HO: Hyper-ovulution
LBW: Low birth weight
NICU: Neonatal Intensive Care Unit
OR: Odds ratio
SGA: Small for gestational age
